# High-energy X-ray diffraction experiment employing a compact synchrotron X-ray source based on inverse Compton scattering

**DOI:** 10.1016/j.zemedi.2024.03.003

**Published:** 2024-04-16

**Authors:** Johannes Melcher, Martin Dierolf, Benedikt Günther, Klaus Achterhold, Daniela Pfeiffer, Franz Pfeiffer

**Affiliations:** a Chair of Biomedical Physics, Physics Department, TUM School of Natural Sciences, Technical University of Munich, 85748 Garching, Germany; b Munich Institute of Biomedical Engineering, Technical University of Munich, Boltzmannstr. 11, 85748 Garching, Germany; c Department of Diagnostic and Interventional Radiology, TUM School of Medicine, Klinikum rechts der Isar, Technical University of Munich, 81675 München, Germany; d TUM Institute for Advanced Study, Technical University of Munich, Lichtenbergstraße 2a, 85748 Garching, Germany

**Keywords:** Munich compact light source, Inverse Compton X-ray source, Kidney stone, X-ray diffraction, Wide-angle X-ray scattering

## Abstract

X-ray diffraction (XRD) is an important material analysis technique with a widespread use of laboratory systems. These systems typically operate at low X-ray energies (from 5 keV to 22 keV) since they rely on the small bandwidth of K-lines like copper. The narrow bandwidth is essential for precise measurements of the crystal structure in these systems. Inverse Compton X-ray source (ICS) could pave the way to XRD at high X-ray energies in a laboratory setting since these sources provide brilliant energy-tunable and partially coherent X-rays. This study demonstrates high-energy XRD at an ICS with strongly absorbing mineralogical samples embedded in soft tissue. A quantitative comparison of the measured XRD patterns with calculations of their expected shapes validates the performance of ICSs for XRD. This analysis was performed for two types of kidney stones of different materials. Since these stones are not isolated in a human body, the influence of the surrounding soft tissue on the XRD pattern is investigated and a correction for this soft tissue contribution is introduced.

## 1 Motivation

X-ray Diffraction (XRD) is a commonly used material investigation technique. It is used for multiple purposes ranging from material determination to chemistry applications and questions in life science.

Typically laboratory XRD systems operate at the K-lines of the anode materials since a narrow bandwidth results in sharper diffraction peaks, which allow for a more straightforward data analysis and peak differentiation. The most common sources are X-ray tubes with an anode typically made from Ag, Mo, Cu, Co or Cr. Their $K_{\alpha }$ lines range from 5 keV in the case of Cr to 22 keV for Ag. This is perfectly suited for research applications in which the sample can be directly mounted onto a sample holder located right in front of the X-ray source. The more challenging case is, an XRD measurement on a sample embedded in soft tissue, which attenuates the X-rays. In these cases, a higher X-ray energy is better suited because the attenuation decreases with a rate of approximately $1/E^{3}$ with increasing X-ray energy. To overcome the decreasing scattering angle, one adjusts the sample to detector distance to maintain the same resolution.

In this article, we investigate the capabilities of inverse Compton X-ray sources for this type of XRD measurement since they generate brilliant X-rays with a narrow bandwidth in a higher energy range than the classical tube sources.

In close cooperation with the Klinikum rechts der Isar, Technical University of Munich, kidney stones were selected as best suited sample. Kidney stones are typically located in the kidneys or in the urinary tract. Information about the composition of kidney stones is important for appropriate therapy. Ideally, this information should be obtained non-invasively and in situ, without the need for an interventional or surgical procedure to remove the stones for diagnosis. Typical imaging modalities employed in clinics include ultrasound [1], X-ray computed tomography (CT) [1], and dual-energy CT [2]. The most common kidney stone components are: calcium oxalate monohydrate (COM), calcium oxalate dihydrate (COD), summarized in calcium oxalate stones, uric acid (UA) and carbonate-apatite (CA).

A few XRD approaches have been developed for the investigation of a stone’s composition, which will be briefly summarised in the following:

A common procedure is to grind extracted kidney stones and examine the resulting powder. This method was used in various publications [3], [4], [5], [6], [7], [8], [9], [10].

Comparisons of different analytic methods on ground kidney stones were performed by several groups, e.g.  Pollack et al. used IR-Absorption Spectroscopy and XRD [11].

Total reflection X-ray fluorescence, wavelength dispersive X-ray fluorescence and XRD were measured by Bielecka et al. [12], furthermore X-ray absorption near-edge spectroscopy and XRD were utilized by Siritapetaweea and Pattanasiriwisawab [13].

Extracted and intact kidney stones were investigated with XRD utilizing a Gd-filtered X-ray beam from a polychromatic X-ray source by Davidson et al. [14], [15], [16]. The stones were individually translated and rotated and a diffraction pattern was acquired at each position to calculate a ”coherent scatter computer tomography (CSCT)”. They resolved the different material compositions spatially, limited by the very long acquisition times to a single slice of the sample.

Furthermore, XRD was also used on intact kidney stones covered with a pig-fat layer at the synchrotron VEPP-3 by Ancharov et al. [17], [18].

This prior work mainly investigated isolated specimens. However, kidney stones are embedded inside tissue in a human. Consequently, the information on the stone’s composition should be acquired before treatment and non-invasively ideally, i.e. without surgical removal of the stone. This may be possible with XRD employing hard X-ray radiation like provided by ICSs.

Various X-ray imaging techniques have already been translated from synchrotrons to inverse Compton X-ray sources, like the Munich Compact Light Source (MuCLS), since they provide synchrotron radiation in a laboratory setting [19]. Due to their narrow natural bandwidth and high spatial as well as spectral flux density, these sources should be highly suitable for XRD experiments. In this study, the results of the proof-of-principle experiments performed at the MuCLS are presented and evaluated.

## 2 Theory

The underlying physics of X-ray diffraction is elastic scattering of the incoming X-rays. Since X-ray scattering theory is well understood, the interested reader is referred to textbooks such as [20], [21].

Suppose an incoming X-ray beam of wavelength λ illuminates a crystal with an atomic plane spacing d at an angle Θ. If this angle is defined between the surface of the crystal and the incoming ray, the angle at which constructive interference occurs can be calculated with the Bragg equation [22]:

(1) n·λ=2dsin(θ)

Here *n* describes the order of the reflection. The atomic plane spacing d can be derived from the lattice constant a and the corresponding Miller indices (h,k,l) for the respective plane.

The complete scattering pattern consists of the sum of the diffraction by the various electron densities in the volume of interest. In poly-crystalline randomly orientated objects like kidney stones, this pattern transforms into an array of concentric rings.

**Figure 1:**
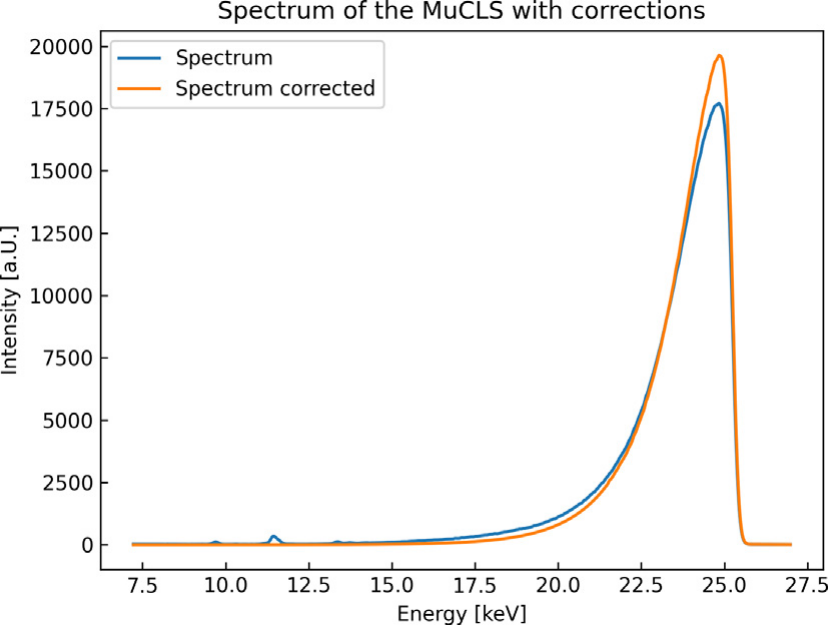
Spectrum of the Munich Compact Light Source (MuCLS) recorded with a silicon drift detector (Ketek AXAS-D, Ketek GmbH, Munich, Germany). The blue curve is the spectrum measured, the orange one is corrected for absorption in the detector and air. The low-energy tail is fitted and replaced by an ideal exponential decay to remove the noise amplified by the correction.The small peaks around 11 keV are the fluorescence background of the tungsten slits.

The material information is encoded in the mean radial position of the rings and their intensity. If one takes a closer look at one individual ring it shows a characteristic radial intensity profile, which is dominated by the source spectrum (see Fig. 1) and contains other factors like the specific beam geometry which are extensively discussed in [23, Chapters 5 and 6].

Since crystals with different compositions produce different diffraction patterns, their composites can be determined from the recorded diffraction rings. Their different intensities are explained by scattering theory, a comprehensive derivation can be found in [23, Chapter 3]. The contribution of different materials to a XRD pattern is discussed in [23], [24]. In essence, the combined pattern is a sum of the single-material XRD patterns weighted according to their concentration in the sample.

Therefore, XRD is extremely well suited for discriminating different types of kidney stones.

## 3 Methods

### 3.1 Calculation of the XRD signal of poly-crystalline objects

To obtain a theoretical basis for the experiments and to get a first impression of the XRD patterns, these are calculated and compared. Calculation of the expected XRD patterns was performed in Python using the functionalities offered by the package xrayutilities [25], [26], [27]. The material parameters for the calculation such as the lattice constants are provided by the Cambridge Crystallographic Data Centre [28]: COM [29], [30], COD [31], [32] and UA [33], [34]. The lattice parameters are listed in supplementary Table 2. Furthermore, the calculation considers the measured X-ray spectrum of the MuCLS depicted in Fig. 1. Its measurement is explained in Section 3.3.

An XRD pattern is calculated for every spectral intensity with the xrayutilities PowderModel function in the 2θ angular range from 0° to 30° [35].

These patterns are afterwards summed up incoherently. The pseudo-code can be found in Algorithm 1.

As the stones usually consist of multiple components, first, the contributions of the individual materials are calculated separately as explained above and then summed up incoherently in a second step.

Algorithm 1 Pseudocode for the calculation algorithm

**Table:** 

import calculate_powder_pattern from xrayutilities
load material from database
from corrected_spectra_measurements load intensity, energy
**for** energy in spectrum **do**
pattern(energy).append(calculate_powder_pattern(material,energy))
**end for**
XRD pattern = sum(pattern,axis=”energy”)

### 3.2 The Munich Compact Light Source

The XRD experiments were performed at the Munich Compact Light Source (MuCLS). Its inverse Compton X-ray source was developed and manufactured by Lyncean Technologies Inc. [36], [37] and upgraded in collaboration with the Technical University of Munich (TUM). The surrounding infrastructure, such as the experimental endstations, was designed and built by TUM staff [38], [19].

**Figure 2:**
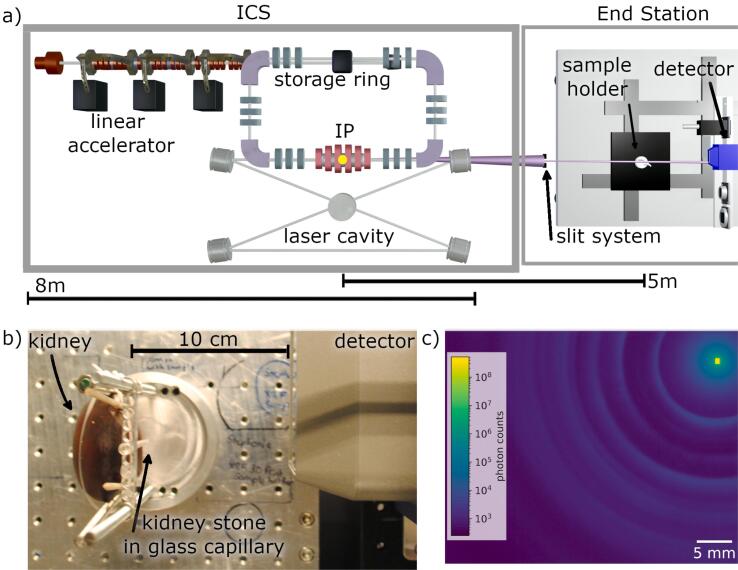
Implementation of X-ray Diffraction (XRD) at the MuCLS. (a) Schematic drawing of the experimental setup. A slit system located ∼3.15 m from the X-ray source(Interaction Point, IP) cuts out a small square X-ray beam of 0.25 mm × 0.25 mm or 1 mm × 1 mm from the full X-ray beam. This pencil beam hits the kidney stone and the kidney. The scattered X-rays are detected with a Pilatus 200K hybrid photon counting detector (Dectris AG, Baden, CH). (b) Photograph of the setup. The kidney stone is contained inside a glass tube located at the back of the kidney in the depicted situation. The detector is placed 10 cm downstream of the kidney stone (5.078 m from IP) (c) A typical detector image from Sample A, created as sum from 2730 single images with a exposure time of 1 s each, acquired at 25 keV with a slit opening of 1 mm^2^.

X-rays are generated by the interaction of relativistic electrons with laser photons in the inverse Compton source. The electrons circulate in a small storage ring and collide with the counter-propagating laser beam circulating in an optical enhancement cavity. This interaction point (IP) is indicated with a yellow circle in Fig. 2. The footprint of the source is 2.5 m × 7 m.

Electron and photon beam parameters as well as confinement of the X-ray beam into a full opening angle of 4 mrad result in a spatially homogeneous X-ray spectrum with a narrow bandwidth of 3% to 5% and an X-ray beam brilliance of 1.2 × 10^10^ph/s/mm^2^/0.1% bandwith [19]. After exiting the ICS, the X-rays propagate in free space to two endstations that provide a flexible infrastructure for a variety of experiments. A comprehensive review of the MuCLS facility can be found in Günther et al. [19].

### 3.3 Measurement of the X-ray spectrum

As the spectrum of the MuCLS is not infinitesimal small, the spectrum of the source has to be taken into account. To later include the source spectrum into the XRD simulations, the spectrum is recorded with a silicon drift detector (Ketek AXAS-D, Ketek GmbH, Munich, Germany). This detector is calibrated with X-ray fluorescence lines of Cr, Fe, Ni and Mo reference samples. Subsequently, the spectrum is smoothed with a running mean and corrected for the absorption in air as well as in the sensor of the detector. As this process increases the noise in the low-energy part of the spectrum, the latter is replaced by an exponential function, which is fitted to the spectrum between 21.51 keV and 23.14 keV. In [39, Chapter 9.2.2] Günther proves the agreement of an exponential function with the decay on the low energy side of the spectrum by comparison with Monte-Carlo simulations. The resulting spectrum is displayed in Fig. 1.

### 3.4 Experimental setup

The XRD experiments presented in the following were conducted at the first endstation of the MuCLS. Fig. 2 a) shows a schematic of the setup. Right after the X-rays enter this endstation, a motorised slit system (Huber Diffraktionstechnik GmbH, Rimsting, DE) confines the X-ray beam of ∼13 mm in diameter to a small square pencil beam of 0.25 mm × 0.25 mm or 1 mm × 1 mm. At a distance of 5.078 m downstream the X-ray source (measured from the IP), this pencil beam impacts on a kidney stone located inside a glass capillary (soda glass, product name “Spezialglas”, WJM Glas Müller GmbH, Berlin, DE) having a wall thickness of 10 μm [40].

The XRD signal of the pure glass capillary is negligible, cf. supplementary Fig. 5, which is in contrast to other potential container materials e.g.  made of plastic. The capillary is mounted onto a holder designed in–house and depicted in Fig. 2 b). This holder is attached to a motorised rotation and translation axis. The capillary itself is aligned with the former’s centre of rotation. To simulate the influence of tissue, a pig kidney can be optionally attached to the capillary. Since the holder can rotate, one can choose to either have the stone or the kidney closer to the detector.

The square pencil beam with an edge-length of 1 mm was used for the measurements of a pure glass capillary, the different kidney stones inside this capillary and the pure kidney, while a smaller pencil beam with an edge-length of 0.25 mm was employed for any combination of kidney stones and kidney. The scattering signal is recorded with a Pilatus 200K Hybrid photon counting detector ([41], Dectris AG, Baden, CH) which is located at a distance of 10 cm downstream the kidney stone. Its pixel size is 172 μm × 172 μm. The angular resolution per pixel of the setup with the used pixel size and distance is 0.1°. With the beam size of 1 mm the resolution is further decreased to 0.6°.

An X-ray energy of 25 keV and acquisition times between 1 s and 30 s were used. Its choice depended on the measurement configuration, i.e.  whether a pure stone or a stone-tissue combination was measured. The criterion was the minimum acquisition time at which the XRD pattern was well recognisable.

To improve statistics, several hundred images were recorded and averaged afterwards. The total number of images per scan ranged from 300 to 1000 to account for variations of the X-ray flux in between 0.78 and 1.33 × 10^10^ ph/s. Fig. 2 c) shows an example of the summed detector images of kidney stone sample A.

### 3.5 Data analysis

The XRD patterns are integrated from the raw data using a custom Python script that performs the four main steps: loading and normalizing, finding the central peak of the XRD pattern, and performing the azimuthal integration with pyFAI [42], [43].

The loaded images are averaged and divided by the flux measured with the MuCLS monitoring system [44] to account for the flux fluctuations between the scans.

The images with different acquisition times are normalised by the corresponding exposure time. An iterative approach is used to find the location of the central spot. It carries out an azimuthal integration of the diffraction pattern with several different positions for the centre and searches for the maximum of the resulting intensities. The procedure is repeated with a higher resolution around the previously found maximum. The detector gap of the Pilatus 200K Detector is considered in every azimuthal integration via the pyFAI Pilatus 200K detector class, which creates a binary mask and considers only detector pixels outside the gap.

The azimuthal integration around the previously determined centre returns the final intensity versus the scattering angle 2Θ.

#### 3.5.1 Correction of superimposed tissue signal

Inside a human, a kidney stone is surrounded by soft tissue whose XRD signal superimposes the signal of the stone.

In order to recover the stone signal, the background contributed by the soft tissue must be known. This can be determined by a reference XRD measurement, cf.  supplementary Fig. 6.

Since the position of the kidney and thus its distance to the detector varies slightly from experiment to experiment due to its rotation from the front to the back position, the sample detector distance is adjusted in the evaluation algorithm. The criterion is the correspondence of the characteristic homogeneous broad plateau of the reference pure kidney XRD pattern to the plateau in the measurement of the kidney stone with the soft tissue.

To compensate for the slightly varying absorption on different spots in the soft tissue of the kidney, the two reference measurements are scaled to the same intensity level, thus the wide tissue peak in both data sets has the same intensity. The final correction is performed by subtracting the homogeneous background signal from the superimposed one. Since the signals of the glass container and scattering in air, cf.  supplementary Fig. 5, are weak compared to the measured signal, they can be neglected. This procedure takes into account the signal of the actual tissue surrounding the kidney stone, i.e.  its specific properties and inhomogeneities, which is more accurate compared to adapting a general model.

### 3.6 Samples

The kidney stone samples, including the stone composition data, is provided by the Klinikum rechts der Isar, Technical University of Munich.

The composition of the kidney stones can be found in Table 1. Further details about the medical study design as well as the selection of patients can be found in [45]. The reference composition is determined by Fourier-transform infrared spectroscopy (FTIR) [46] using a Spectrum 100 system (Perkin Elmer, Beaconsfield, UK) comparing the recorded spectra with databases implemented in the Spectrum 100 software. The diameter of the samples varies from 3 mm to 8 mm. For each category, two stones were measured. The measured data was averaged for each pair.

**Table 1:** Overview of the relative composition of the seven samples used in the experiment. The table’s top row specifies both, the chemical compound name as well as the mineral name, for every sample.

	Composition [%]		
compound Name	UA	COM	COD
mineralogical name	Uricite	Whewellite	Weddellite
Sample A		90	10
Sample B	50	50	

## 4 Results & discussion

The result section is structured in increasing complexity of the measurement task. To verify the experimental results, the obtained measurements were compared with the calculated XRD patterns first in section  4.1. The comparison was performed with both uric acid-based and calcium-oxalate-based kidney stones.

Afterwards, the influence of soft tissue, in this case a pig kidney, on the recorded XRD pattern was investigated. Finally, an approach to correct for this soft tissue XRD background is presented in Section 4.1.1.

### 4.1 Measurement of kidney stones

**Figure 3:**
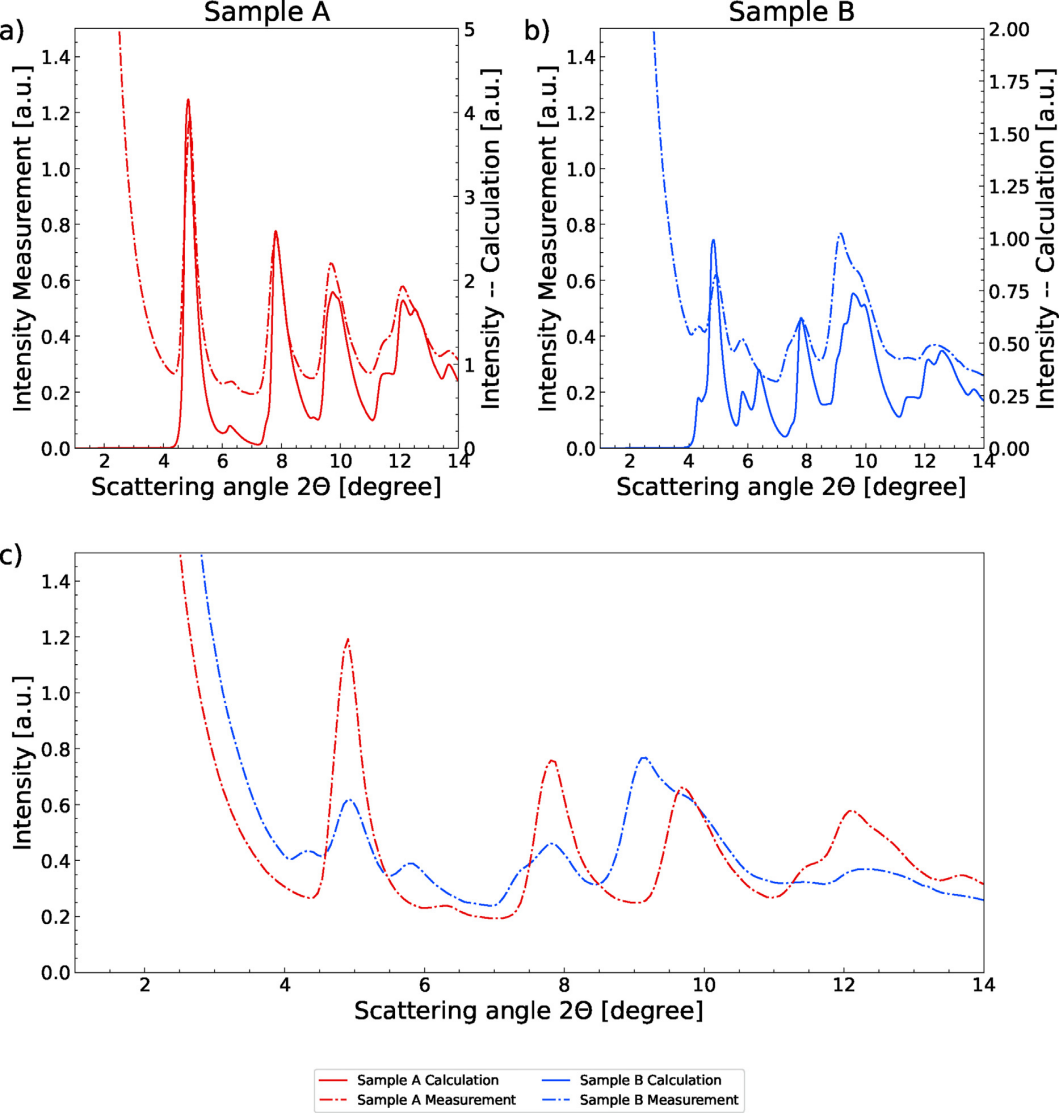
Comparison of calculation (solid line) with measured data (dashed dotted line) for a calcium oxalate stone (sample A) displayed in (a) and a uric acid stone type (sample B) depicted in (b). A comparison between measurements of the samples A and B is shown in (c).

The first step is to compare the calculation of the XRD pattern with the measured ones. Fig. 3 a) and b) display the calculated intensities of Samples A and B as well as the measurements of the corresponding stones. For a better visual perception of differences and similarities between the two measured XRD patterns, they are displayed in the same plot in Fig. 3 c).

In sample B (half UA), the peaks expected around 4.9°, 7.8°, 9.1° and 12.3° from the calculation match the measured data very well. Besides the peak height of the 9.1° peak, the height and position of the peaks agree in calculation and measurement. For the kidney stone consisting of COM (sample A), Fig. 3 a), the calculated and measured peak heights and positions at 4.6°, 7.5°, 9.3° and 11.7° agree even better. The finer structure like the double peaks in the simulation of Sample B could not resolved in the experimental data due to the limitation of the angular resolution by the used beam size. This also causes the remaining background between the peaks.

The difference between the uric acid and COM stones can be easily seen in Fig. 3 c). The peaks variation in intensity and their slightly different angular distribution enable discrimination of UA and COM.

#### 4.1.1 Measurement with a kidney

The soft tissue surrounding a kidney stone contributes a broad background intensity to the scattering signal [47], [48]. Furthermore, the radiation scattered by the stone is partially absorbed inside the tissue. Both effects reduce the scattering signal’s intensity level compared to the background. To investigate these effects, porcine kidney tissue was first measured without and subsequently with a kidney stone.

The diffraction pattern of a pure porcine kidney can be found in the Supplementary Fig. 6. It features a smooth and wide pattern with a higher plateau around 10°. Paterno et al. observed a similar shape in their investigations [48].

**Figure 4:**
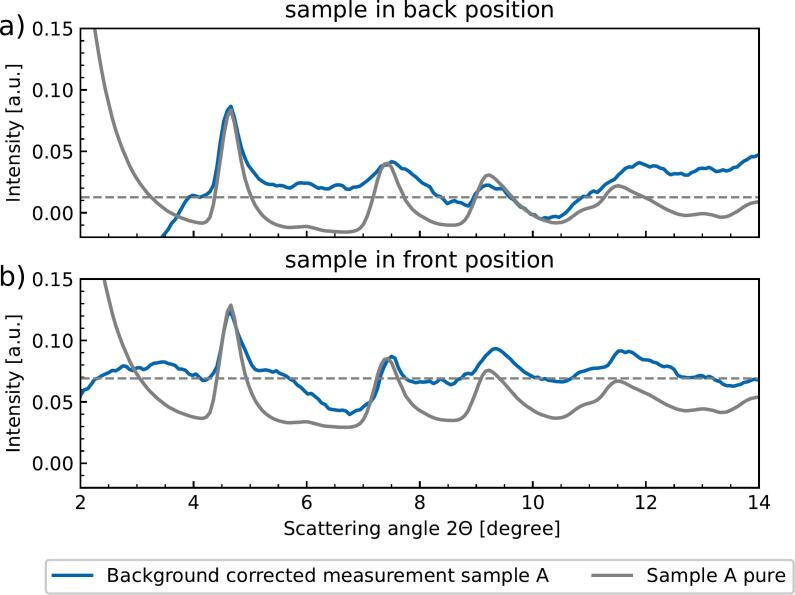
XRD Pattern of Sample A when it is placed behind (a) or in front (b) of a pig kidney. Both measurements are corrected for the homogeneous background (see Section 3.5.1 (blue curve). The XRD measurement of the pure stone is included in gray color in both subplots for comparison (scaled and shifted). The dashed line is the base level (background level) for peak comparison.

To examine a kidney stone located on the outer surface of the kidney between the kidney and the detector, sample A was placed behind the kidney (“back position”). Additionally, the sample holder can rotate. This way the kidney stone can be moved in front of the kidney and the kidney is in between the stone and detector (“front position”). In this way, the two maximum cases can be achieved: either the beam has to pass through the entire kidney and is diffracted, or the beam hits the stone, is diffracted and then passes through the kidney. The recorded raw data was analysed according to Section 3.5.1 and is depicted in Fig. 4 including the correction for the continuous kidney background. For comparison, the pure signal of sample A is included as well.

In the back position, the first three prominent peaks of the pure stone’s diffraction pattern correlate in angular position with the three peaks of the stone in the presence of soft tissue. The decreasing maximum peak height of the pure stone data could also be represented in this measurement for the first three peaks. The increase in intensity at an angle larger than 10° originates from the subtraction of the scattering signal from the soft tissue of the kidney, as the homogeneous scattering signal differs a bit from this angle on. Nevertheless, the fourth peak can still be recognized.

In the front position, the location of the first four peaks matches the pure measurement.

The fourth peak could be seen as a broader peak whose shape is similar to the pure one.

At larger scattering angles, the intensity decreases due to the subtraction method. Consequently, the characteristic features of the COM-stone are maintained in the presence of soft-tissue independent of the stone’s exact position inside the soft tissue.

Comparing the amplitude of the first diffraction peak of both measurements, the peak’s amplitude relative to the background (0.046) measured at the front position is about 30% weaker than the one (0.069) measured at the back position.The base line for the amplitude measurement relative to the background is the dashed plotted line in Fig. 4. This corresponds to the theoretically calculated intensity difference, calculated by the Beer–Lambert law with an absorption coefficient for soft-tissue [49], [50] and a sample thickness of 2.5 cm.

Sample B was measured in similar conditions and the results can be found in supplementary Fig. 7.

## 5 Conclusion and outlook

This study presents the first proof-of-principle XRD experiments for the discrimination of different common types of kidney stones with an inverse Compton source. The benefits of the MuCLS are its partially coherent quasi-monochromatic, i.e.  synchrotron-like, X-ray radiation at higher photon energies than the ones provided by the K-lines of common X-ray tubes.

The higher availability of an inverse Compton X-ray source compared to large-scale synchrotrons facilitates collaboration with a university hospital without scheduling constraints.

Both, calculation and experimental validation demonstrate the capabilities of this method.

Even in the presence of organic tissue, the scattering pattern could be retrieved using a reference measurement. However, absorption within the tissue reduces the scattering signal’s intensity which in turn affects the quality of the retrieved data.

The absorption of several soft tissue layers around the kidney, will reduce even more the signal intensity. Further work is needed to improve the signal quality, for example by using a photon counting detector with smaller pixel size and measure more than one position in the kidney stone.

The location of the stone has to be precisely targeted. One option would be a X-ray radiograph of the kidney. To minimize potential influence of stone motions, the time between imaging and XRD should be as short as possible. Another possible implementation in clinical practice would be to use ultrasound to identify the location of the kidney stone and then to record the XRD pattern with a targeted X-ray beam. As the stone is tracked, motion artefacts could be taken into account. In addition to improving the XRD acquisition speed, an ultrasound transceiver compatible with the XRD-acquisition geometry must be developed. In particular, this requires a robust matching of the ultrasound and X-ray coordinate systems. Dual-energy CT has the advantage of being an already approved medical examination that works reliably. However, this techniques exposes an extended abdominal volume to ionising radiation while XRD would apply dose only very localised to the region of interest.

The X-ray energy used in the experiments is 25 keV at which the absorption in tissue is stronger than at higher X-ray energies. For example, at 50 keV the attenuation coefficient for soft tissue [50] is about 54 % lower, which for a thickness of 5 cm translates to an 3.7 times higher transmitted intensity.

The elastic scattering cross section of COM at 50 keV in contrast is about 32 % from the one at 25 keV. This is an advantage if the examination involves more surrounding tissue.

The decreasing scattering angle could then be overcome either technically by using a detector with a smaller pixel size and the same geometry or geometrically by moving the same detector further away.

For future studies with tissue, a higher X-ray energy would be more suitable, which is an opportunity for the next generation of inverse Compton sources that can produce higher energies.

## 6 Data statement

Raw data were generated at MuCLS. Derived data supporting the results of this study are available on reasonable request from the corresponding author Johannes Melcher.

## 7 Ethics

The samples in our measurements were acquired by the Klinikum rechts der Isar, Technical University of Munich. Each patient had their renal stone(s) removed following the common clinical practice with respect to their individual diagnosis and indication. For this retrospective analysis, informed consent was waived by the local ethics committee. The porcine kidney used for the experiments in Section 4.1.1 was bought by the local butchery.

## 8 CRediT author statement

Johannes Melcher: Methodology, Software, Formal analysis, Investigation, Data Curation, Writing - Original Draft, Visualization;

Martin Dierolf: Investigation, Conceptualization, Writing - Review & Editing;

Benedikt Günther: Writing - Review & Editing;

Klaus Achterhold: Writing - Review & Editing, Project administration;

Daniela Pfeiffer: Conceptualization, Resources;

Franz Pfeiffer: Funding acquisition, Conceptualization, Supervision, Resources.

## Declaration of Competing Interest

The authors declare that they have no known competing financial interests or personal relationships that could have appeared to influence the work reported in this paper.
