# Supplementary material for: High-energy X-ray diffraction experiment employing a compact synchrotron X-ray source based on inverse Compton scattering
Source: Z Med Phys. 2024 Apr 16;35(4):428–37. doi: 10.1016/j.zemedi.2024.03.003 (PMC12766491; doi:10.1016/j.zemedi.2024.03.003)
Supplement: Supplementary Data 1 [file mmc1.pdf]

8. Supplementary Material

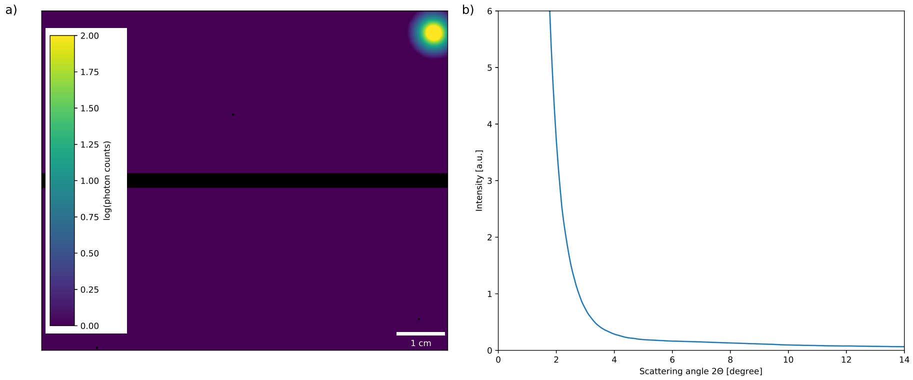

Figure 5: Measurement of an empty glass container in air a) averaged detector image displayed in a logarithmic colour code. b) resulting 2 Θ-Plot of the glass capillary. The slit size here is 1 mm^2, the number of exposures amounts 1000 with 1 s exposure time

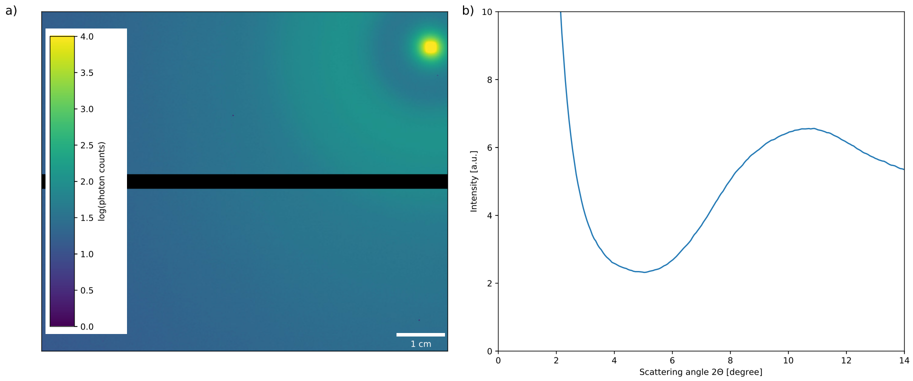

Figure 6: Measurement of a pure kidney: a) averaged detector image displayed in a logarithmic colour code. The black stripe originates from the detector module gap of the used Pilatus 200K detector. In part b) the result of the azimuthal integration is shown.The slit size here is 1 mm^2 the number of exposures amounts 15 with 30 s exposure time

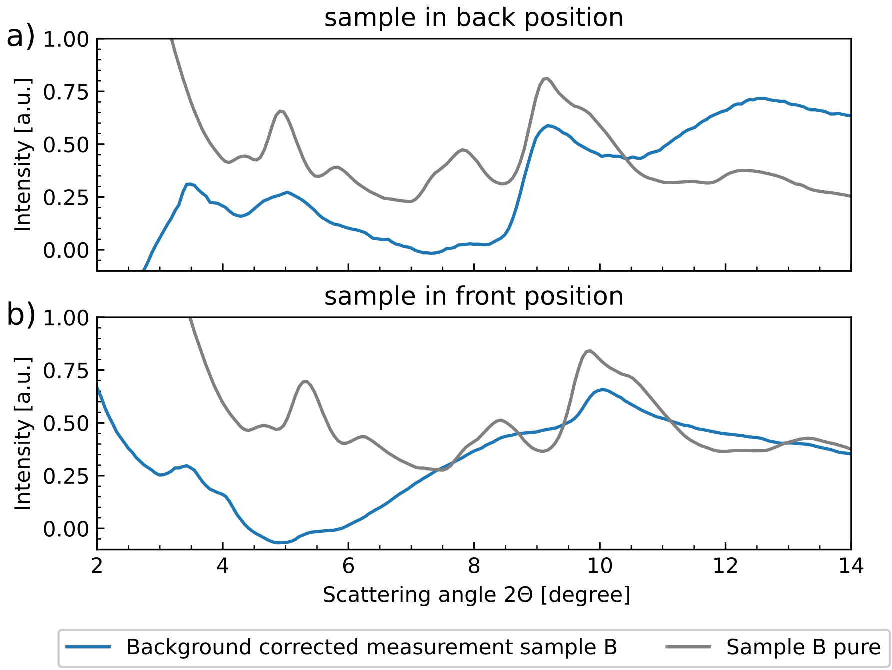

Figure 7: XRD Pattern of Sample B when it is placed behind (a) or in front (b) of a pig kidney. Both measurements are corrected for the homogeneous background (see section 3.5.1 (blue curve). The XRD measurement of the pure stone is included in gray color in both subplots for comparison (scaled and shifted). The slit size is 0.5 mm × 0.5 mm, resulting in higher intensities as in Figure 4.

	**COM**	**COD**	**UA**
a[Å]	6.290	12.378	6.2352
b[Å]	14.583	12.378	7.2756
c[Å]	10.116	7.389	13.1328
α [°]	90	90	90
β [°]	109.46	90	90.710
γ [°]	90	90	90

Table 2: Most important lattice parameters for the used calculation of the materials COM [29, 30], COD [31, 32] and UA [33, 34]. All parameters are given for the unit cell.
